# Analysis of Factors Contributing to Adverse Events and Evaluation of Their Impact on Prognosis in Metastatic Renal Cell Carcinoma Patients—Real-World Experience in a Single-Center Retrospective Study and Narrative Review

**DOI:** 10.3390/medicina60030398

**Published:** 2024-02-26

**Authors:** Piotr Domański, Mateusz Piętak, Szymon Staneta, Weronika Fortuniak, Barbara Kruczyk, Adam Kobiernik, Piotr Bakuła, Anna Mydlak, Tomasz Demkow, Bożena Sikora-Kupis, Paulina Dumnicka, Jakub Kucharz

**Affiliations:** 1 Department of Experimental Immunotherapy, Maria Sklodowska-Curie National Research Institute of Oncology Roentgena 5, 02-781 Warsaw, Poland; 2 Department of Genitourinary Oncology, Maria Sklodowska-Curie National Research Institute of Oncology Roentgena 5, 02-781 Warsaw, Poland; mateusz.pietak98@gmail.com (M.P.);; 3 Department of Head and Neck Oncology, Maria Sklodowska-Curie National Research Institute of Oncology Roentgena 5, 02-781 Warsaw, Poland; 4 Chair of Biochemistry, Jagiellonian University Medical College, 31-034 Kraków, Poland

**Keywords:** clear cell renal cell carcinoma (ccRCC), cabozantinib, adverse events (AEs), prognostic factors, Tyrosine Kinase Inhibitors (TKIs)

## Abstract

*Background and Objectives:* More than 430,000 new cases of renal cell carcinoma (RCC) were reported in 2020. Clear cell RCC, which occurs in 80% of cases, is often associated with mutations in the VHL gene, leading to dysregulation of hypoxia-induced transcription factors pathways and carcinogenesis. The purpose of this study is to examine the adverse events (AEs) of cabozantinib treatment and the relationship between individual patient factors and the frequency of their occurrence in detail. *Materials and Methods:* Seventy-one patients with metastatic RCC were treated with second or further lines of cabozantinib at the Department of Genitourinary Oncology, Maria Sklodowska-Curie National Research Institute of Oncology. Comprehensive data, including demographics, clinicopathological factors, and AEs, were collected from January 2017 to June 2021. This study evaluated the impact of various patient-related factors on the rate of adverse events and treatment tolerance using a Cox proportional hazards model. *Results:* Cabozantinib-induced AEs were significantly associated with body mass index (BMI), body surface area (BSA), IMDC prognostic score, and treatment line. Notably, patients receiving cabozantinib post-tyrosine kinase inhibitors reported fewer AEs. Dose reduction was unrelated to adverse event frequency, but patients requiring dose reduction were characterized with lower body mass and BSA but not BMI. *Conclusions:* The factors described make it possible to predict the incidence of AEs, which allows for faster detection and easier management, especially in the high-risk group. AEs should be reported in detail in real-world studies, as their occurrence has a significant impact on prognosis.

## 1. Introduction

In the global population, renal cell carcinoma (RCC) ranks as the 9th most common cancer in men and the 14th most common in women. In 2020 alone, there were more than 430,000 new cases of kidney cancer. The incidence of this cancer shows significant geographical variation, with a higher prevalence observed in northeastern Europe [1]. In Poland, RCC constitutes approximately 3.8% of all cancers, leading to around 6500 annual diagnoses. About 2800 people succumb to kidney cancer each year in Poland, contributing to 2.8% of all cancer-related deaths [2].

The predominant subtype is clear cell renal cell carcinoma (ccRCC), with up to 80% exhibiting a mutation in the VHL gene. The VHL protein’s role in degrading hypoxia-induced transcription factors (HIF-1α and HIF-2α) is crucial. Dysfunction in VHL leads to the accumulation of HIF1 factors, triggering the overexpression of growth factors such as vascular endothelial growth factor (VEGF) and fostering carcinogenesis [3,4].

While age is the most significant risk factor, smoking, obesity, and hypertension also significantly increase the risk of RCC. The median diagnosis age is 64 years old, varying with geographical location. Early detection through improved access to ultrasound allows for kidney-sparing treatment [5]. Nevertheless, 30% of new diagnoses are metastatic, and 20% of patients experience recurrence or metastasis after radical treatment, necessitating systemic treatment [6].

The past decade has seen a significant prolongation of overall survival in patients with metastatic clear-cell renal cell carcinoma. Advances in therapy have been made possible by improved treatment regimens and the introduction of new tyrosine kinase inhibitors and immunotherapies. Both ESMO [7] and ASCO [8] recommend the use of a combination of tyrosine kinase inhibitors (TKIs) with immune checkpoint inhibitors (ICIs) as the first-line treatment for patients with metastatic clear cell carcinoma, regardless of prognostic group, proving highly active and effective therapy and resulting in objective response rates as high as 55.7% [9]

Multikinase inhibitors like cabozantinib inhibiting MET, AXL, VEGFR, and other kinases and receptors are commonly used. VEGFR inhibition suppresses angiogenesis, increasing hypoxia within the tumor and limiting further growth. Research indicates that cabozantinib exhibits immunomodulatory properties by diminishing the infiltration of suppressor cells, including regulatory T cells and myeloid-derived suppressor cells (MDSCs), within the tumor [10,11]. Additionally, it directly affects tumor cells, increasing their sensitivity to the cytotoxic effects of the immune system. This might not be the sole mechanism for amplifying the efficiency of the cellular response, potentially leading to increased effectiveness of immunotherapy [12].

The second element of the aforementioned combination is ICIs. The backbone of their action is the prevention of T-cell anergy and exhaustion, resulting in their activation and effective anti-tumor response. This process involves blocking the fusion of the PD-1 receptor on T cells with PD-L1 present on tumor cells and activated T cells [13]. However, it is acknowledged that the interaction of PD-1 with PD-L1 is not the only mechanism by which T lymphocytes can be inhibited. Additionally, as T cells undergo activation, PD-L1 expression increases. Similarly, PD-L1 shows a significant rise in depleted CD4+ T cells [14], which may secondarily inhibit the anti-tumor response and lead to the depletion of activated T cells. 

The purpose of this study is to examine the adverse events (AEs) and their relationship with individual patient factors and the frequency of their occurrence in detail. In the treatment of ccRCC with TKIs, AEs such as hand-foot syndrome (HFS), hypothyroidism, or diarrhea have been identified as valuable predictive factors, associated with improved overall survival (OS) and progression-free survival (PFS) [15,16,17,18]. One must bear in mind that cabozantinib was often used as a second-line drug; more geriatric patients may be in this group compared to those receiving first-line drugs, necessitating adjustments in dosing [19]. The study also aims to highlight patients particularly vulnerable to AEs, allowing clinicians to pay closer attention to facilitate more effective management.

## 2. Materials and Methods

### 2.1. Patient Collection

This retrospective analysis included seventy-one patients with biopsy-proven metastatic clear cell renal cell carcinoma (mRCC) undergoing cabozantinib treatment as a second or subsequent line at the Department of Genitourinary Oncology of the Maria Skłodowska-Curie National Research Institute of Oncology in Warsaw.

The database contained the data of patients with mRCC treated at the department between 30 January 2017 and 23 June 2021. This study was conducted following the principles of the Declaration of Helsinki. Permission to conduct this study was granted by the Maria Sklodowska-Curie National Research Institute of Oncology Bioethics Committee (permission number 38/2018).

### 2.2. Data Collection and Inclusion Criteria

The database contained detailed information on age, gender, clinicopathological factors, laboratory results, comorbidities, adverse events, sites of metastases, ECOG performance score, International Metastatic RCC Database Consortium (IMDC), and Memorial Sloan-Kettering Cancer Center (MSKCC) risk scores [20], along with outcome data associated with individual patients. Clinical data were extracted from medical records, and mortality data were obtained from the Polish national database. Detailed characteristics of the study group at the study’s commencement are shown in Table 1.

The study included patients aged between 42 and 80 years with biopsy-proven metastatic clear-cell renal cancer. The initial dose of cabozantinib for all patients was 60 mg per day. Dose modifications were based on the Summary of Product Characteristics [21]. Complete blood counts (CBC) were evaluated for each patient before starting the course of treatment with cabozantinib. Hematological parameters were measured using Sysmex XN-1000 (Aleje Jerozolimskie 176, Warsaw, Poland), and laboratory tests were conducted by the Diagnostic Department of the National Research Institute of Oncology. Patients were classified into three risk groups (favorable, intermediate, and poor) for both MSKCC and IMDC based on their scores, but none of these groups were excluded from the study. Adverse events were assessed following the Common Terminology Criteria for Adverse Events (CTCAE) v5.0 [22]. Table 2 displays the previous lines of treatment.

This study included patients eligible for systemic treatment with biopsy-proven metastatic ccRCC. Patients with non-clear cell renal cell carcinoma were also included if they received cabozantinib through emergency access to drug technologies due to the failure of other treatment options. Moreover, cabozantinib had to be administered as a second- or subsequent-line treatment with an initial dose of 60 mg per day. Consent to treatment and participation in the study were also required. 

### 2.3. Assessment of Treatment Response and Adverse Events

Response to treatment was assessed by examination and computed tomography (or MRI) scan every 12 weeks. All imaging data were assessed by radiologists with experience in tumor response evaluation, which was conducted according to the RECIST criteria [23]. Responses were confirmed after three months of treatment with cabozantinib. Adverse events were assessed following the Common Terminology Criteria for Adverse Events (CTCAE) v5.0 [22]. Patients were duly informed of all potential AEs and were actively encouraged to provide details of any changes in their well-being or the onset of new complaints or symptoms. Subsequently, AEs were thoroughly assessed at follow-up appointments, which were scheduled every two weeks. Treatment persisted until either disease progression or the onset of significant toxicity, classified as Grade 4 (G4). Any necessary adjustments to the dosage were carefully deliberated in collaboration with the patient, following a comprehensive benefit-risk assessment. The collected data are summarized in Table 3.

### 2.4. Search Strategy and Selection Criteria

Publications of interest were English-language reports of real-world studies (RWEs) or randomized clinical trials (RCTs) of cabozantinib use in patients with RCC. Eligible papers for the table had to include insightful information about cabozantinib-related AEs and dosage reduction. A search of PubMed was conducted (20 December 2023) covering the period from 2012 to 2023. Search criteria were as follows: ((cabozantinib) AND (RCC or Renal cell carcinoma)) NOT (combined or nivolumab). The search yielded 222 papers, but only 51 of them were considered consistent with the topic of this paper were retrieved from PubMed. Then, we analyzed in detail and finally included information from 27 [15,18,24,25,26,27,28,29,30,31,32,33,34,35,36,37,38,39,40,41,42,43,44,45,46,47,48] publications that were considered useful for this manuscript, while the remaining references came from the personal knowledge of the authors [49,50]. In the end, we qualified 13 [15,26,27,28,29,31,33,34,35,42,44,51,52] works to formulate a table describing AEs; in the remaining 14 papers, information about AEs was insufficient. The search and preliminary selection of papers was performed by two of the authors (P.D. and W.F.). Subsequently, all authors reviewed and checked the inclusion and exclusion criteria and the data of the resulting manuscripts, which all authors agreed to include in this paper.

### 2.5. Statistical Analysis

Categorical variables were summarized with the number and percentage of the respective group. Quantitative variables were summarized with mean and standard deviation (SD; normally distributed) or median and first and third quartile (Q1; Q3; non-normally distributed) as specified in Results. The subgroups were compared with Pearson chi-squared test, Fisher exact test (2 × 2 tables, expected frequency < 5), *t*-test, or Mann-Whitney test, respectively. Spearman rank correlation coefficients were used to evaluate the associations between the number of reported adverse events during cabozantinib treatment and patients’ characteristics. All the statistical tests were two-tailed, and the results were interpreted as significant at *p* < 0.05. Statistica software (version 13; Tibco, Tulsa, OK, USA) was used for computations.

## 3. Results

**Figure 1:**
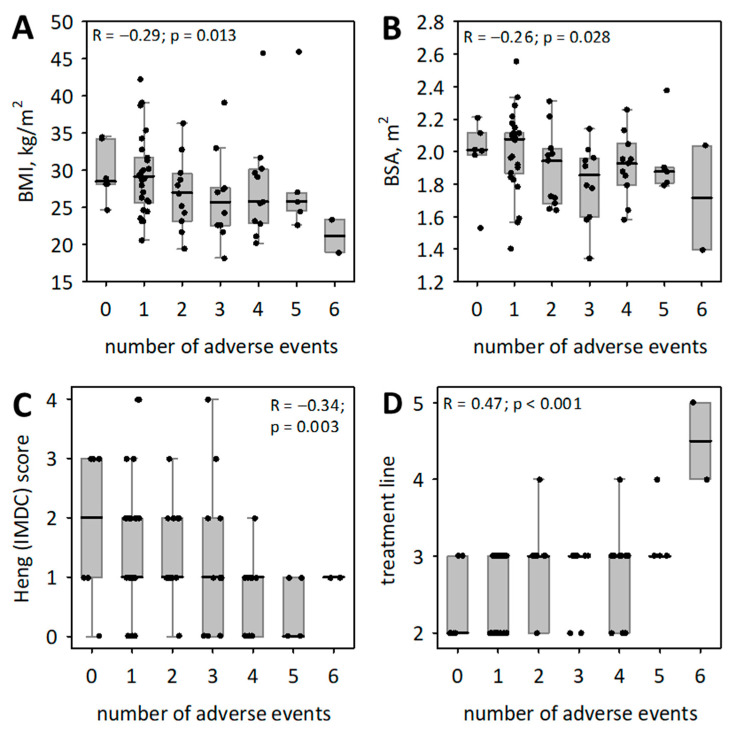
Statistically significant associations between the patients’ characteristics and the number of cabozantinib adverse events. Data are shown as median (middle bar), interquartile range (box), non-outlier range (whiskers); points represent raw data. Spearman rank correlation coefficients and the respective *p*-values are shown on the graphs. (**A**)—Correlation of BMI and number of adverse events. (**B**)—Correlation of BSA and number of adverse events. (**C**)—Correlation of IMDC score and number of adverse events. (**D**)—Correlation of treatment line and number of adverse events.

The number of adverse events related to cabozantinib significantly correlated with BMI, BSA, IMDC prognostic score, and the cabozantinib treatment line, as illustrated in Figure 1. Moreover, a correlation was observed with body mass (R = −0.28; *p* = 0.020). Age, sex, or median time from diagnosis were not associated with the number of adverse events. Patients who received cabozantinib directly after tyrosine kinase inhibitors (TKI), regardless of the treatment line, reported a lower number of distinct adverse events: median (Q1; Q3) equaled 1 (1; 3) versus 3 (2; 4); *p* = 0.032. Furthermore, among patients treated with cabozantinib in third or further line, those who received TKI in second-line treatment (*n* = 20) experienced a lower number of adverse events: 2 (1; 3) than those who received everolimus or temsirolimus in second line (*n* = 18): 4 (3; 5); *p* < 0.001. These associations were held when comparing patients with 0–1 adverse events to those with more than 1 adverse event.

There was no association between the number of reported adverse events and the need for cabozantinib dose reduction (*p* = 0.2). Among the adverse events, only hypertension was more prevalent in patients requiring dose reduction (64% vs. 36%; *p* = 0.041). Moreover, patients requiring dose reduction were characterized with lower body mass [mean (SD): 77.1 (19.1) versus 87.1 (18.4) kg; *p* = 0.028] and BSA [1.85 (0.25) vs. 2.01 (0.21) m^2^; *p* = 0.010], but not BMI. Dose reduction was significantly more common in women (80% vs. 33%; *p* < 0.001). There was no association between dose reduction and age, treatment line, time from diagnosis, or other baseline characteristics of patients, or with previous treatment. 

The number of cabozantinib adverse events was associated with BMI, BSA, IMDC prognostic score, and cabozantinib treatment line (Figure 1); it also correlated with body mass (R = −0.28; *p* = 0.020). Age, sex, or median time from diagnosis were not associated with the number of adverse events. Patients who received cabozantinib directly after tyrosine kinase inhibitors (TKI), irrespective of treatment line, reported a lower number of distinct adverse events: median (Q1; Q3) equaled 1 (1; 3) versus 3 (2; 4); *p* = 0.032. Moreover, among patients treated with cabozantinib in third or further line, those who received TKI in second-line treatment (*n* = 20) had a lower number of adverse events: 2 (1; 3) than those who received everolimus or temsirolimus in second line (*n* = 18): 4 (3; 5); *p* < 0.001. All the above associations were confirmed when we compared patients with 0–1 and more than 1 adverse event.

There was no association between the number of reported adverse events and the need for cabozantinib dose reduction (*p* = 0.2). Among the adverse events, only hypertension was more prevalent in patients requiring dose reduction (64% vs. 36%; *p* = 0.041). Moreover, patients who needed dose reduction were characterized with lower body mass [mean (SD): 77.1 (19.1) vs. 87.1 (18.4) kg; *p* = 0.028] and BSA [1.85 (0.25) vs. 2.01 (0.21) m^2^; *p* = 0.010], but not BMI. Dose reduction was much more common in women (80% vs. 33%; *p* < 0.001). There was no association between dose reduction and age, treatment line, time from diagnosis, or other baseline characteristics of patients, or with previous treatment. 

## 4. Discussion

### 4.1. Interpretation of Results Based on Analysis of Existing Literature

The study showed that lower scores according to IMDC criteria correlate with a higher incidence of AEs. Furthermore, the findings illustrated in Figure 1D emphasize a consistent pattern: the more advanced the line of cabozantinib treatment, the higher the incidence of AEs. This phenomenon could potentially be attributed to the robust therapeutic activity of cabozantinib in the further stages of RCC treatment [53]. Alternatively, it might be associated with the likelihood that patients undergoing subsequent lines of treatment are more susceptible to frailty syndrome, thereby elevating the risk of AEs. Recent investigations into RCC have indicated no correlation or have even suggested that obese cancer patients may have a lower risk of mortality compared to their normal-weight counterparts [54]. This is known as the obesity paradox. Nishihara et al. found that the inverse links between BMI and mortality among some disease subgroups may be due to the high heterogeneity of the disease [55]. The longer survival among obese patients could be due to a less aggressive disease subtype [56,57] and also the fact, that obesity is associated with a higher incidence of clear cell histology than other histological types [58,59]. We observed a noteworthy correlation between higher BMI and BSA and a reduced incidence of AEs.

### 4.2. Mini Review of Cabozantinib-Related AEs in Randomized Clinical Trials and Real-World Experiences

After a comprehensive literature review, we have opted to prepare a Table 4 showing cabozantinib-related AEs. We emphasize the noteworthy distinctions observed between the reporting of AEs in RCTs and RWEs. We are aware of all the difficulties associated with accurate data collection in day-to-day work. However, this indicates good communication with patients and the ability to collect much-needed information. The identified studies unanimously confirm that hypothyroidism, HFS, diarrhea, and hypertension are the most prevalent cabozantinib-related AEs. This aspect holds crucial implications for the overall treatment landscape, as many of these AEs may serve as predictive factors [16,17,18,60,61]. Effective management of a substantial number of AEs poses challenges for both clinicians and patients. The increased occurrence of AEs can hinder patient compliance, and it is crucial to recognize that each AE carries the potential for severe, even life-threatening complications. However, it is important to acknowledge that AEs also serve as indicators of the therapy’s efficacy, with some being anticipated outcomes in the treatment process.

The correlation between AEs and prognosis has been observed in many systemic therapies—starting with chemotherapy, followed by TKIs and ending with immunotherapy [16,17,18,62,63]. In studies involving RCC patients, AEs occurred as potential predictive factors across various ongoing treatments [60,61,63,64]. Notably, early onset of hypertension or its worsening during ongoing sunitinib therapy has been identified as a favorable factor in advanced stages of the RCC and can be considered as a predictive factor for PFS and ORR [61]. Additionally, the manifestation of HFS during sunitinib treatment was noticed to improve OS and PFS [60]. Verzoni et al. observed a prolonged OS in patients receiving nivolumab after experiencing any type of AEs [64]. This underscores their potential role as valuable indicators in guiding therapeutic strategies for enhanced efficacy of treatment, especially in RCC.

Similar associations have been observed across various cancer types. Chen et al. and Zhou et al. reported that immune-related adverse events (irAEs) served as a positive predictive factor for PFS in non-small cell cancer lung patients undergoing treatment with anti-PD1 inhibitors as a subsequent line of therapy [62,63]. Additionally, similar potential in this prognosis has also been found by blood markers like PLR, NLR, SII [65], and CRP levels [66]. Nevertheless, more studies need to be done in this field to understand the correlation between onset of AEs and prediction of prognosis as an underlying mechanism of above mentioned interplay; despite some hypothesis, this still remains unknown [63,64,67].

### 4.3. Efficacy of Cabozantinib and the Search for Synergistic Combines

Many studies have confirmed the efficacy of cabozantinib in RCC. To begin with, the METEOR trial compared the efficacy of cabozantinib versus everolimus in second-line treatment aRCC and showed that cabozantinib achieves better results in terms of median OS and PFS (21.4 months vs. 16.5 months and 7.4 months vs. 3.8 months, respectively) [33]. These results were consistent with many real-world retrospective studies such as CERES, in which the ranges of OS and PFS were 6.01 and 10.84 months, respectively. Nevertheless, the median PFS in the CERES study was shorter, which can be explained by the fact that 99% of patients were treated at stage IV [47]. Median PFS for patients treated with cabozantinib in the Polish Managed Access Program (MAP) and Italian MAP were 12.5 months and 8 months, respectively [15,30]. The CABOSUN compared the efficacy of sunitinib verus cabozantinib as first-line treatment in the poor risk group and proved cabozantinib to improve outcomes in terms of PFS and OS (8.2 months [95% CI, 6.2 to 8.8 months] versus 5.6 months [95% CI, 3.4 to 8.1 months] and 30.3 months [95% CI, 14.6 to 35.0 months] versus 21.8 months [95% CI, 16.3 to 27.0 months], respectively [31]. Chun Loo Gan et al. conducted a retrospective study examining the effectiveness of cabozantinib therapy from first to subsequent lines of treatment. The ORR, time to treatment failure (TTF), and OS of patients treated with cabozantinib in the first to fourth lines of therapy were comparable; that was also reported in our previous study [53]. It is noteworthy that across all lines of therapy, the TTF and mOS were significantly longer for patients who required dose reduction versus. patients who did not, with an adjusted HR of 0.37 (95% CI 0.202–0.672, *p* < 0.01) and 0.46 (95% CI 0.215–0.980, *p* = 0.04), respectively. There was no difference in the ORR in patients with or without dose reduction (23% vs. 26%, *p* = 0.69) [32].

Cabozantinib emerged as an effective therapy for treating bone metastases in patients with prostate cancer, prompting researchers to conduct a subgroup analysis within the METEOR trial, which included patients with or without bone metastases. PFS for patients with bone metastases was 7.4 months (95% CI, 5.5 to 10.4 months) for patients treated with cabozantinib versus 2.7 (HR, 0.33 [95% CI, 0.21 to 0.51]) for patients treated with everolimus. Among patients with bone metastases, ORR evaluated by the Independent Radiology Committee was 17%, whereas no ORR was observed with everolimus. Additionally, the median OS for patients treated with cabozantinib was 20.1 months, compared to 12.1 months for those treated with everolimus. However, it is worth mentioning that the METEOR study was not powered for statistical testing of subgroup analysis [68].

The combination of ICIs with VEGFR inhibitiors (VEGFRi) has emerged as a significant step forward in cancer treatment. A synergistic effect has been observed between these two classes of drugs, contributing to improved outcomes across various studies, with a continually expanding number of registered clinical trials. The combination of cabozantinib with nivolumab in aRCC therapy was compared with sunitinib in the CheckMate 9ER trial—this novel approach achieved better results in terms of PFS and probability of OS at 12 months (16.6 months [95% CI 12.5 to 24.9] versus 8.3 months [95% CI, 7.0 to 9.7] and 85.7% [95% CI, 81.3 to 89.1] versus 75.6% [95% CI, 70.5 to 80.0], respectively [9]. A systemic review by Niewada et al. compared the combination of cabozantinib with nivolumab to other ICI-VEGFRi or ICI-ICI combinations. This review revealed no statistically significant differences in terms of OS in cabozantinib + nivolumab and any other combination [69]. 

The CANTATA trial, which compared a glutaminase inhibitor telaglenastat + cabozantinib versus placebo + cabozantinib, confirmed the effectiveness of cabozantinib in the population that was previously treated with IPI-NIVO. There was no improvement in the antitumor activity of cabozantinib in the telaglenastat + cabozantinib group [70]. 

### 4.4. The Synergism Phenomenon of Combining VEGFR Inhibitors with Immune Checkpoint Inhibitors

Through VEGFR inhibition, cabozantinib not only provides an antiangiogenic effect [71], limiting a tumor’s growth potential [72], but also demonstrates notable immunomodulatory properties. The accumulation of VEGF within the tumor inhibits ICAM-1 and VCAM-1 expression on endothelial cells, potentially impeding immune cell infiltration [73]. Simultaneously, it promotes lymphocytes CD8+ apoptosis while increasing Treg infiltration [74]. Moreover, it contributes to the myeloid-derived suppressor cells (MDSCs) maturation [75] and inhibits the maturation of dendritic cells (DCs) [76,77]. TKIs have the potential to counteract these effects: they alter the relationship between effector and regulatory cells [12], thus creating an immunostimulatory environment [78,79] and enhancing tumor infiltration through normalization of vascularization [80]. Additionally, they restore DC maturation [81] and reduce the number of MDSCs [12]

In mouse models, cabozantinib significantly decreased the expression of PD-L1 on renal cancer cells via c-Met signaling inhibition [82]; a similar effect is hypothesized to occur through AXL inhibition [53].

Taken together, these effects indicate that cabozantinib not only influences the tumor microenvironment but also directly modifies the tumor cells, making them more vulnerable to immune-mediated destruction [12], thus supporting the use of TKI plus immunotherapy combinations in oncology.

### 4.5. Difficult Treatment Choice after Progression

The latest treatment guidelines form ESMO [7] and ASCO [8] recommend highly active VEGFRi-ICIs treatment as the first-line choice in eligible patients. Despite its tremendous activity, there is a group of patients who will not respond or will progress during treatment with VEGFRi-ICIs. This raises the question of second-line treatment following VEGFRi-ICIs failure. While some prospective and retrospective studies have explored the outcomes of using VEGFR TKIs, such as pazopanib, sunitinib, or cabozantinib, in post-immunotherapy regimens, it is important to note that these studies often involve small and heterogeneous patient cohorts. Powles T.B. et al. reported a mPFS of 7.4 months in a group of patients receiving pazopanib after the failure of the ICI-based treatment regimens [83]. The INMUNOSUN-SOGUG trial by E. Grande et al. aimed to assess the activity of sunitinib as a second-line treatment in patients pre-treated with ICI-based regimens. The study failed to reach the pre-specified endpoint (i.e., a 30% ORR). The relatively short mPFS of 5.6 months may suggest that possibly a different choice of VEGFRi-TKI should be considered [84]. McGregor et al. explored the activity of cabozantinib in the same setting. The study included 86 patients, out of which 29% were pre-treated with VEGFRi-ICIs combination. The median TTF was 6.5 months and the mOS was 13.1 months [52]. The possibility of further usage of ICIs in the second line of treatment after the failure of VEGFRi-ICIs is still a subject of research lacking sufficient evidence and is not included in the ESMO guidelines [7]. Moreover, a combined therapy with cabozantinib and belzutifan, an HIF-2α inhibitor, is also currently being researched. Choueiri et al. examined the efficacy of belzutifan + cabozantinib as a subsequent line after previous immunotherapy. In the intervention arm, 16 of 52 (31%) patients had a confirmed objective response: one (2%) complete response and 15 (29%) partial responses, showing promise as a subsequent line of therapy for this patient group, though further investigation is needed [85].

### 4.6. Limitations

Limitations of this study include the small size of the subgroups and the retrospective nature of the research. Additionally, the paper is a narrative review. A detailed systematic review with meta-analysis would provide results with a lower risk of bias. A single-center study allows for uniform and well-defined criteria, but we are aware that a multicenter study would significantly increase the number of patients included in the study and the reliability of the study. Further studies should be conducted to assess the other correlations between individual factors such as BMI, BSA, IMDC prognostic score, or treatment line and adverse events. Uncovering and reporting these correlations can contribute to providing more informed and personalized patient care. Our findings, therefore, serve as a valuable reference point for future real-world studies focusing on metastatic renal cell carcinoma (mRCC).

## 5. Conclusions

Factors like IMDC score, BMI, and BSA may be useful tools in the day-to-day prediction of AEs risk in mRCC patients. That, in turn, will allow for faster detection and easier management, especially in the high-risk group. Profile and intensity of AEs may be equivalent of treatment response, and their careful interpretation can contribute to achieving better therapeutic outcomes. Further improvement in knowledge of AEs and more frequent reporting of them in RWEs studies is needed. This study showed that reporting accuracy in RWEs is comparable to RCTs, yet the vast majority of papers omit this clinically relevant aspect.

## Figures and Tables

**Table 1:** Characteristics of studied group at the start of the study (=start of cabozantinib treatment).

Characteristic	Values Observed in mRCC Patients (*n* = 71)
Male sex, *n* (%)	46 (65)
Mean age (SD), years	63 (9)
Median time from RCC diagnosis (Q1; Q3), years	4.3 (2.0; 8.2)
Mean body weight (SD), kg	82.1 (19.3)
Mean BMI (SD), kg/m^2^	28.1 (5.9)
Mean body surface area (BSA) (SD), m^2^	1.93 (0.24)
Morphology	
Clear cell, *n* (%)	69 (97)
Non-clear cell, *n* (%)	6 (8)
Sarcomatoid differentiation, *n* (%)	11 (14)
Nephrectomy, *n* (%)	69 (97)
Fuhrman grade	
1, *n* (%)	6 (8)
2, *n* (%)	33 (46)
3, *n* (%)	21 (30)
4, *n* (%)	11 (15)
MSKCC score	
0, *n* (%)	19 (27)
1, *n* (%)	36 (51)
2, *n* (%)	15 (21)
3, *n* (%)	1 (1)
IMDC prognostic score	
0, *n* (%)	16 (23)
1, *n* (%)	30 (42)
2, *n* (%)	15 (21)
3, *n* (%)	7 (10)
4, *n* (%)	3 (4)
Metastases	
Lungs, *n* (%)	53 (75)
Bone, *n* (%)	24 (34)
Liver, *n* (%)	12 (17)
Pancreas, *n* (%)	6 (8)
Other sites, *n* (%)	31 (44)
Median number of sites (Q1; Q3)	2 (2; 3)
ECOG performance score	
0, *n* (%)	19 (27)
1, *n* (%)	42 (59)
2, *n* (%)	9 (13)
3, *n* (%)	1 (1)
Karnofsky performance scale	
100, *n* (%)	10 (14)
90, *n* (%)	21 (30)
80, *n* (%)	37 (52)
<80, *n* (%)	3 (4)
Cabozantinib as second-line treatment, *n* (%)	30 (42)
Cabozantinib as third-line treatment, *n* (%)	36 (50)
Cabozantinib as fourth- or fifth-line treatment, *n* (%)	5 (7)

**Table 2:** Previous treatment (before the initiation of cabozantinib).

	*n* (%)
First-line treatment	
TKI (sunitinib, pazopanib, sorafenib), *n* (%)	65 (92)
Other (immunotherapy), *n* (%)	6 (8)
Second-line treatment	
TKI (axitinib, sunitinib, pazopanib, sorafenib), *n* (%)	20 (28)
Everolimus, temsirolimus, *n* (%)	18 (25)
Nivolumab, *n* (%)	3 (4)
Third-line treatment	
TKI (sorafenib, pazopanib), *n* (%)	4 (6)
Nivolumab, *n* (%)	1 (1)
Fourth-line treatment (nivolumab), *n* (%)	1 (1)

**Table 3:** Adverse events observed in patients and the need for dose reduction.

Variable	Values Observed in mRCC Patients (*n* = 71)
Any adverse event, *n* (%)	65 (92)
Hypothyroidism, *n* (%)	35 (49)
Hand-foot syndrome, *n* (%)	33 (46)
Hypertension, *n* (%)	28 (39)
Diarrhea, *n* (%)	28 (39)
Asthenia, *n* (%)	24 (34)
Liver toxicity, *n* (%)	11 (15)
>1 reported adverse event, *n* (%)	39 (55)
Median number of adverse events (Q1, Q3)	2 (1–4)
Dose reduction	35 (49)

**Table 4:** Cabozantinib-related adverse events in previous studies.

Author (Year)	Patients, n Male n (%) Female *n* (%)	Mean Age (SD), Years	ECOG Performance Score, n (%)	IMDC Score, *n* (%)	CTAE All Stages, *n* (%)	CTAE ≥ 3, *n* (%)	Dose Reduction, *n* (%)
Domański et al. (2024) [59]	71	63 (9)	0, *n* = 19 (27) 1, *n* = 42 (59) 2, *n* = 9 (13) 3, *n* = 1 (1)	0, *n* = 16 (23) 1, *n* = 30 (42) 2, *n* = 15 (21) 3, *n* = 7 (10) 4, *n* = 3 (4)	Hypothyroidism, *n* = 35 (49) Diarrhea, *n* = 28 (39) HFS, *n* = 33 (46) Hypertension, *n* = 28 (39) Any AEs: *n* = 65 (92)	N/D	35 (49)
Patients treated in clinical trials							
METEOR Choueiri et al. (2016) [33]	330, male, *n* = 253 (77) female, *n* = 77 (23)	63	0, *n* = 226 (68) 1, *n* = 104 (32)	Favorable, *n* = 66 (20) Intermediate, *n* = 210 (64) Poor, *n* = 54 (16)	Hypothyroidism, *n* = 76 (23) Diarrhea, *n* = 249 (75) HFS, *n* = 142 (43) Hypertension, *n* = 122 (37) Any AEs: *n* = 331 (100)	Hypothyroidism, *n* = 0 Diarrhea, *n* = 43 (13) HFS, *n* = 27 (8) Hypertension, *n* = 49 (15) Any AEs: *n* = 235 (71)	206 (62)
CABOSUN Choueiri et al. (2017) [31]	79, male, *n* = 66 (84) female, *n* = 13 (16)	63	0, *n* = 36 (46) 1, *n* = 33 (42) 2, *n* = 10 (13)	Intermediate, *n* = 64 (81) Poor, *n* = 15 (19)	Hypothyroidism, *n* = 18 (23) Diarrhea, *n* = 57 (73) HFS, *n* = 33 (43) Hypertension, *n* = 52 (67) Any AEs: *n* = 75 (96)	Hypothyroidism, *n* = 0 Diarrhea, *n* = 8 (10) HFS, *n* = 6 (8) Hypertension, *n* = 22 (28) Any AEs: *n* = 53 (68)	36 (46)
Retrospective cohort and real-world experience studies							
Prisciandaro et al. (2019) [29]	17 male, *n* = 11 (65) female, *n* = 6 (35)	66	0, *n* = 3 (18) 1, *n* = 12 (70) 2, *n* = 2 (12)	Favorable, *n* = 9 (53) Intermediate, *n* = 8 (47) Poor, *n* = 0	Hypothyroidism, *n* = 4 (24) Diarrhea, *n* = 6 (35) HFS, *n* = 4 (24) Hypertension, *n* = 3 (18) Any AEs: *n* = 16 (94)	Hypothyroidism, *n* = 1 (6) Diarrhea, *n* = 2 (11) HFS, *n* = 0 Hypertension, *n* = 3 (18) Any AEs: *n* = 7 (41)	8 (47)
Lemke et al. (2019) [44]	38 male, *n* = 25 (66) female, *n* = 13 (34)	58	0, *n* = 2 (5) 1, *n* = 21 (55) 2, *n* = 15 (40)	Favorable, *n* = 7 (18) Intermediate, *n* = 20 (56) Poor, *n* = 9 (26)	Hypothyroidism, *n* = 13 (34) Diarrhea, *n* = 11 (29) HFS, *n* = 13 (34) Hypertension, *n* = 9 (24) Any AEs: N/D	Hypothyroidism, *n* = 4 (24) Diarrhea, *n* = 0 HFS, *n* = 1 (3) Hypertension, *n* = 0 Any AEs: N/D	22 (58)
McElwee et al. (2019) [26]	35 male, *n* = 30 (86) female, *n* = 5 (14)	64	0, *n* = 8 (23) 1, *n* = 20 (57) 2, *n* = 7 (20)	N/D	Hypothyroidism, no data Diarrhea, N/D HFS, *n* = 9 (26) Hypertension, *n* = 8 (23) Any AEs: N/D	N/D	8 (23)
Gomez de Liano Lista et al. (2018) [51]	128 male, *n* = 87 (68) female, *n* = 41 (32)	62	0, *n* = 20 (16) 1, *n* = 85 (66) 2, *n* = 23 (18)	Favorable, *n* = 35 (27) Intermediate, *n* = 62 (49) Poor, *n* = 27 (21) Unknown, *n* = 4 (3)	N/D	Hypothyroidism, N/D Diarrhea, *n* = 12 (9) HFS, *n* = 6 (5) Hypertension, N/D Any AEs: 48 (37)	N/D
Bodnar et al. (2019) [15]	115 male *n* = 84 (73) female, *n* = 31 (27)	64	0, *n* = 19 (16) 1, *n* = 79 (69) 2, *n* = 17 (15)	Favorable, *n* = 14 (12) Intermediate, *n* = 79 (69) Poor, *n* = 22 (19)	Hypothyroidism, *n* = 77 (67) Diarrhea, *n* = 70 (61) HFS, *n* = 52 (45) Hypertension, *n* = 51 (44) Any AEs: *n* = 115 (100)	Hypothyroidism, *n* = 0 Diarrhea, *n* = 11 (10) HFS, *n* = 14 (12) Hypertension, *n* = 6 (5) Any AEs: *n* = 56 (49)	79 (69)
McGregor et al. (2020) [52]	86 male *n* = 61 (71) female, *n* = 25 (29)	63	N/D	Favorable, *n* = 4 (5) Intermediate, *n* = 57 (66) Poor, *n* = 24 (28)	Hypothyroidism, N/D Diarrhea, *n* = 9 (10) HFS, *n* = 14 (16) Hypertension, *n* = 3 (3) Any AEs: *n* = 81 (94)	Hypothyroidism, N/D Diarrhea, *n* = 1 (1) HFS, *n* = 2 (2) Hypertension, *n* = 2 (2) Any AEs: *n* = 20 (23)	39 (45)
Sumanta K. Pal et al. (2021) [42]	44 male *n* = 36 (82) female *n* = 8 (18)	65	N/D	Favorable, *n* = 10 (23) Intermediate, *n* = 28 (64) Poor, *n* = 6 (14)	Hypothyroidism, N/D Diarrhea, *n* = 24 (55) HFS, *n* = 21 (48) Hypertension, *n* = 28 (65) Any AEs: *n* = 42 (97)	Hypothyroidism, N/D Diarrhea, *n* = 2 (4) HFS, *n* = 9 (20) Hypertension, *n* = 14 (32) Any AEs: *n* = 32 (74)	10 (23) *
Matthew T. Campbell et al. (2018) [28]	30 male *n* = 26 (87) female *n* = 4 (13)	58	N/D	Favorable, *n* = 2 (7) Intermediate, *n* = 23 (77) Poor, *n* = 5 (17)	Hypothyroidism, N/D Diarrhea, *n* = 17 (57) HFS, *n* = 11 (37) Hypertension, N/D Any AEs: N/D	Hypothyroidism, N/D Diarrhea, *n* = 0 HFS, *n* = 2 (7) Hypertension, N/D Any AEs: N/D	17 (57)
Nieves Martínez Chanzá et al. (2019) [27]	112 male *n* = 85 (76) female *n* = 27 (24)	60	0, *n* = 23 (20) 1, *n* = 59 (53) 2–3, *n* = 11 (10)	Favorable, *n* = 9 (8) Intermediate, *n* = 71 (63) Poor, *n* = 29 (26) unknown, *n* = 3 (3)	Hypothyroidism, *n* = 17 (15) Diarrhea, *n* = 38 (34) HFS, *n* = 35 (31) Hypertension, *n* = 31 (28) Any AEs: N/D	Hypothyroidism **, *n* = 0 Diarrhea **, *n* = 3 (3) HFS **, *n* = 5 (4) Hypertension **, *n* = 4 (4) Any AEs **: N/D	51 (46)
Noboru Nakaigawa et al. (2023) [34]	35 male, N/D female, N/D	63	N/D	Favorable, *n* = 6 (17) Intermediate, *n* = 22 (63) Poor, *n* = 7 (20)	Hypothyroidism, *n* = 6 (17) Diarrhea, *n* = 24 (67) HFS, *n* = 23 (66) Hypertension, *n* = 15 (43) Any AEs: 35 (100)	Hypothyroidism, *n* = 0 Diarrhea, *n* = 3 (9) HFS, *n* = 3 (9) Hypertension, *n* = 5 (14) Any AEs: 29 (83)	33 (94)
Koji Iinuma et al. (2022) [35]	53 male *n* = 44 (83) female *n* = 9 (17)	72	0, *n* = 18 (34) 1, *n* = 17 (32) 2, *n* = 9 (17) 3, *n* = 8 (15) 4, *n* = 1 (2)	Favorable, *n* = 12 (23) Intermediate, *n* = 17 (51) Poor, *n* = 14 (26)	Hypothyroidism, *n* = 9 (17)) Diarrhea, *n* = 14 (26) HFS, *n* = 9 (17) Hypertension, *n* = 11 (21) Any AEs: 42 (79)	Hypothyroidism, *n* = 0 Diarrhea, *n* = 2 (4) HFS, *n* = 3 (6) Hypertension, *n* = 1 (2) Any AEs: 10 (19)	4 (8) *

N/D—no data; * discontinuation of treatment; ** includes only AEs CTAE 3.

## Data Availability

The data presented in this study are available on request from the corresponding author (accurately indicate status).
